# Can Phosphate-Solubilizing Microorganisms Unlock the Path to Sustainable Amazonian Forest Restoration?

**DOI:** 10.1007/s00284-026-04971-6

**Published:** 2026-05-27

**Authors:** Sidilene Pereira Alves dos Santos, Adamir da Rocha Nina Junior, Sabrina Silva de Oliveira Pinho, Josiane Celerino de Carvalho, Caris dos Santos Viana, Karen Cristina Pires da Costa, Érica Mangaravite, Andreia Varmes Fernandes, Mateus Ferreira Santana, Cleverson Diniz Teixeira Freitas, José Francisco de Carvalho Gonçalves

**Affiliations:** 1 Plant Physiology and Biochemistry Laboratory, National Institute of Amazonian Research - MCTI-INPA, Manaus, AM 69060-020 Brazil; 2 Federal Institute of Education, Science and Technology of Amazonas-IFAM, Humaitá, Rodovia BR230, KM 07, Humaitá, AM 69800-000 Brazil; 3 Faculty of Agronomy, Institute of Agricultural and Regional Development Studies (IEDAR), Federal University of Southern and Southeastern Pará (UNIFESSPA), Folha 31, Quadra 07, Nova Marabá, Marabá, PA 68507-590 Brazil; 4 Department of Microbiology, Federal University of Viçosa-UFV, Viçosa, MG 36570-900 Brazil; 5 Department of Biochemistry and Molecular Biology, Federal University of Ceará, Fortaleza, CE 60440-554 Brazil

## Abstract

Phosphorus (P) availability is a critical constraint for plant growth in tropical ecosystems, primarily due to its low mobility and high fixation by soil colloids. The use of phosphate-solubilizing microorganisms (PSMs) is a sustainable strategy to enhance phosphorus bioavailability, particularly in highly weathered and nutrient-depleted soils. These microorganisms, including bacteria, fungi, actinobacteria (Phylum Actinomycetota), and cyanobacteria, convert insoluble phosphorus into plant-available forms through biochemical pathways such as organic acid production, enzyme secretion, and siderophore release. Although widely studied in agricultural systems, the role of PSMs in forest ecosystems, especially in the Amazon, remains poorly explored. This review synthesizes current knowledge on the diversity, solubilization mechanisms, and applications of PSMs, emphasizing their potential to improve seedling establishment and promote ecosystem resilience in forest restoration efforts. The integration of microbial biofertilization into silvicultural and agroforestry practices may reduce dependence on chemical fertilizers, mitigate environmental impacts, and restore soil fertility in degraded Amazonian landscapes. We underscore the urgent need for further research on locally adapted PSM strains and their functional interactions with native tree species. Ultimately, PSMs represent a promising biotechnological tool for restoring the structure and function of tropical forest ecosystems.

## Introduction

Phosphorus (P) is one of the most important macronutrients in the soil and is essential for plant growth and development [1]. It plays a pivotal role in vital energy transfer processes such as photosynthesis, respiration, and is a structural component of molecules including adenosine triphosphate (ATP), nucleic acids (DNA and RNA), phospholipids in cell membranes and other organic compounds, therefore, essential to plant growth and fitness [2–4].

In natural systems, plants absorb phosphorus primarily through their roots in the form of inorganic phosphate ions (PO₄^3^⁻ ion) dissolved in the soil solution, and it is subsequently transported throughout the plant via the xylem [5]. However, phosphorus mobility in soils is extremely restricted due to its strong binding to minerals such as calcium, iron, and aluminum, which leads to its immobilization in unavailable forms [6]. Phosphorus in the soil can also become unavailable due to its adsorption by clay minerals. Among these, phyllosilicates stand out, as they act as natural reservoirs for phosphorus adsorption, in addition to minerals such as kaolinite, illite, and montmorillonite [6, 7]. Only 1.00–2.50% of P is available in natural soil [6]. Already in crops, it is estimated that only around 20% of the phosphate fertilizers applied are effectively utilized. At the same time, the remainder becomes fixed in the soil matrix or is taken up by microbial biomass [8].

Limited efficiency, resulting from the low natural availability of phosphorus in the soil and reduced absorption of applied fertilizers, causes substantial nutrient losses and high economic costs for agriculture [9]. In this context, bacteria, especially those belonging to the *Actinobacteria* group, and fungi, capable of solubilizing phosphate have gained attention because they act as phosphate-solubilizing microorganisms (PSMs), an ecological and sustainable alternative to improve phosphorus availability and reduce dependence on synthetic fertilizers [10]. These microorganisms increase the bioavailability of phosphorus in the soil by producing organic acids, enzymes, and other metabolites that solubilize unavailable forms of phosphorus [11].

Although the use of PSMs is well-documented in annual crops such as maize, wheat, soybean, and rice, studies involving forest species remain scarce. For instance, the inoculation of phosphate-solubilizing bacteria in wheat significantly increased the labile phosphorus fraction in the soil by over 122% [12]; while the combined inoculation of *Bacillus subtilis* and *Azospirillum brasilense* improved phosphorus uptake in maize [13].

These findings highlight the potential of PSMs to enhance nutrient availability and promote plant productivity. Nevertheless, there is still a knowledge gap that limits our understanding of how these microorganisms interact with tree species, particularly in the Amazon biome.

Recent studies have shown that soil microorganisms are capable of converting insoluble phosphorus in the soil into available forms that can be directly absorbed by plants [14]. In the Amazonian context, understanding the interactions between PSMs and native tree species is essential for developing reforestation strategies, such as planting systems and species selection [15], since these microorganisms can naturally make P available to plants, thereby reducing the use of phosphate fertilizers.

This review focuses on the role of PSMs in forest restoration, with a particular emphasis challenges and possibilities of their application in Amazonian forest species. We thoroughly examine the essential characteristics of these microorganisms and establish their potential as effective alternatives to chemical fertilizers for producing high-quality forest seedlings.

To compile this review, a systematic literature search was conducted in the Scopus, Science Direct, Wiley Online Library, PubMed, Google Scholar, SciELO, and Web of Science databases. The search utilized the following keywords: “microorganisms,” “Amazonian forest species,” and “phosphorus solubilization mechanisms.” Studies published primarily in the last ten years were selected, focusing on the interface between microbial biotechnology and the restoration of Amazonian ecosystems, with the aim of synthesizing the main advances and gaps regarding the interaction between phosphate-solubilizing microorganisms and tropical tree species.

## Phosphorus in the Soil

Soils are known for the spatial distribution of their resources, such as nutrients and water. The topsoil layer, known as the litter, is rich in organic P due to the continuous deposition of plant and animal residues [16]. Plants and microorganisms play a central role in the soil biological activity, significantly influencing P dynamics at the soil-root interface [17]. The bioavailability of P is largely mediated by both organic and inorganic compounds, including mucilage, organic acids, phosphatases, and specific signaling substances [18].

**Fig. 1:**
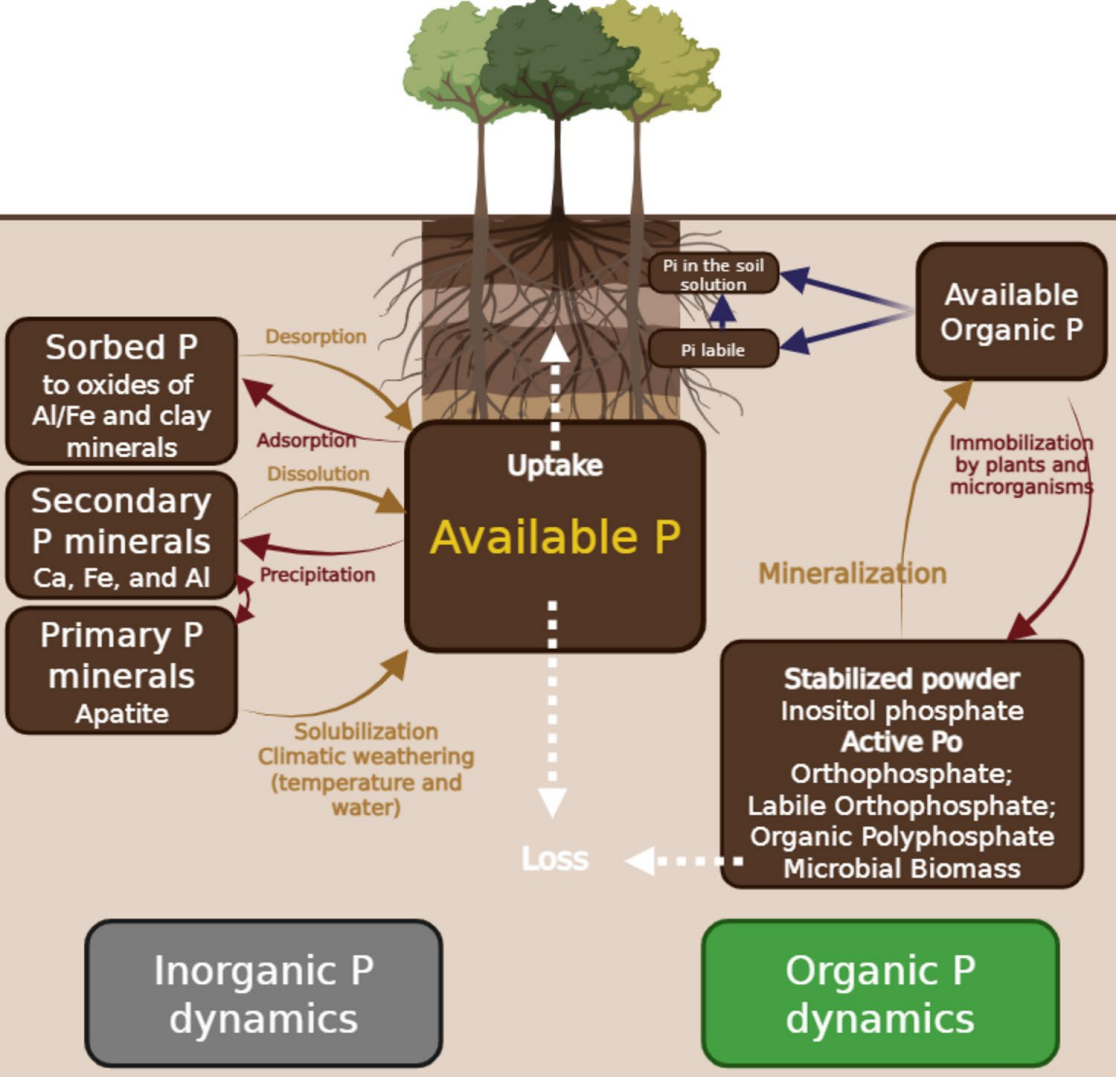
Schematic representation of phosphorus (P) dynamics in soil. Red arrows indicate geochemical and biogeochemical transformations, gold arrows highlight processes mediated by phosphate-solubilizing microorganisms (PSMs), and white arrows represent P fluxes between soil and plants. Abbreviations: P = phosphorus; Pi = inorganic phosphorus; Po = organic phosphorus; Al = aluminum; Ca = calcium; Fe = iron. Adapted from Tian et al., 2023. Created in https://BioRender.com

This element undergoes processes of adsorption, precipitation, mineralization, immobilization, and diffusion in the soil. It is strongly retained by the particles, forming insoluble compounds that are poorly available to plants [19]. Mineralization of organic matter releases inorganic phosphorus, while immobilization converts it back into organic forms. As phosphorus has low mobility, its movement to the roots occurs mainly by diffusion, which limits its availability (Fig. 1).

These processes involve two interconnected compartments: the soil solution and the solid phase, the latter acting as a reservoir that replenishes the nutrient content of the solution. This exchange mechanism is referred to as the capacity factor, which reflects the soil’s buffering ability of phosphorus, a property influenced by the strength of P binding to the solid phase [20].

## Phosphate-Solubilizing Microorganisms (PSMs)

The PSMs include a diverse group, most of bacteria and fungi, capable of converting insoluble phosphorus compounds in the soil into available forms to plants [4]. These microorganisms play a key role in improving phosphorus bioavailability in low-fertility soils, especially those with a high capacity to retain phosphorus in insoluble complexes. Table 1 summarizes the main studies with microbial groups involved in phosphate solubilization, along with their common habitats and main mechanisms of action.

**Table 1:** Main microbial groups of PSMs, their genera, common habitat, main mechanisms of action

Microbial group	Genera	Common habitat	Main mechanism of action	Reference
Phosphate-solubilizing bacteria (PSB)	*Bacillus*, *Pseudomonas*, *Rhizobium*, *Enterobacter*	Rhizosphere of agricultural plants	Organic acids, phosphatases, siderophores	[21]
Phosphate-solubilizing fungi (PSF)	*Aspergillus*, *Penicillium*, *Trichoderma*, *Fusarium*	Organic soil and rhizosphere	Intense secretion of organic acids and phytases	[22]
Phosphate-solubilizing Actinobacteria (PSA)	*Streptomyces, Micromonospora*, *Thermobifida*	Tropical soils and rhizosphere	Enzymes, siderophores, stress resistance	[23]
Cyanobacteria/ Microalgae	*Calothrix*, *Anabaena*	Moist soil, rice rhizosphere	Organic P mineralization and gradual release	[24]

Numerous microorganisms have demonstrated the ability to solubilize otherwise unavailable phosphorus in soils, thereby enhancing its accessibility to plants [25]. Among bacteria, *Bacillus* and *Pseudomonas* species are commonly isolated from the rhizosphere and are known for producing a variety of organic acids and siderophores [4]. Fungal genera, such as *Aspergillus* and *Penicillium,* dominate in organic-matter rich soils and contribute to phosphorus solubilization through the excretion of organic acids such as citric and oxalic acids [26]. Actinobacteria, particularly *Streptomyces*, are gaining recognition for their enzymatic versatility and adaptation to tropical soils [27].

The soil microbiome plays a crucial role in ecosystem functioning through its involvement in nutrient cycling, primary productivity, bioremediation, and plant growth promotion [28]. Microorganisms such as bacteria, fungi, actinobacteria, algae, and protozoa find favorable conditions for development in the soil environment [29]. The use of PSBs as eco-friendly biofertilizers is currently concentrated in South America and Asia, where their role as a complement to or replacement for synthetic phosphate fertilizers has gained increasing attention [30].

Currently, more than 2,700 microbial strains from at least 336 bacterial species, 88 fungal species, and nine archaeal species have been identified as potential phosphate solubilizers [31]. Overall, PSMs offer an effective strategy for mobilizing phosphorus in soils, helping to mitigate the effects of phosphorus deficiency in agriculture. This approach reduces the reliance on chemical fertilizers, whose long-term environmental impacts are well-documented, and contributes to the development of more sustainable and resilient agroecosystems.

### Use of Phosphate-Solubilizing Bacteria (PSBs)

The study on phosphorus-solubilizing bacteria (PSBs) began in the twentieth century, focusing on the biological mechanisms by which these microorganisms increase phosphorus availability to plants. One of the first commercial products, known as Alinit, was developed from a *Bacillus* strain, and its application resulted in an approximately 40% increase in crop yield [32].

The importance of PSBs is also reflected in their widespread occurrence and diversity in soils. It is estimated that PSBs account for between 1 and 50% of the total soil microbial population, depending on soil type and conditions [33]. The genera most frequently studied for their metabolic plasticity and efficiency in secreting organic acids, which are primary agents responsible for phosphorus solubilization in the rhizosphere, include *Pseudomonas*, *Bacillus*, *Rhizobium*, *Serratia*, *Rhodococcus*, and *Enterobacter* [34, 35].

Many studies conducted on agricultural crops have shown that the use of PSBs improves phosphorus nutrition for plants, as these microorganisms are capable of solubilizing organic and inorganic phosphorus in the soil [34–36]. However, in the Amazonian context, evidence regarding these processes is scarce. Endophytic bacteria isolated from the roots of *Bertholletia excelsa*, in native forests and agroforestry systems, have high potential to promote plant growth. These isolates, belonging to genera such as *Bacillus*, are capable of solubilizing complex phosphorus sources, especially aluminum phosphate (AlPO₄), a predominant form of inorganic phosphorus (P) in highly weathered tropical soils [37].

In addition, research on agroforestry systems involving two native Amazonian species, *Theobroma cacao* and *Euterpe oleracea,* has shown that rhizosphere bacteria have demonstrated high potential for the mineralization of organic phosphorus. In this study, of the 48 bacterial strains isolated, 29% and 8% were capable of solubilizing organic and inorganic phosphate, respectively. These results highlight the importance of microbial enzymatic activity in the phosphorus cycle derived from soil organic matter [37].

The use of phosphate-solubilizing bacteria offers significant potential for reducing dependence on chemical phosphate fertilizers. Their documented efficiency across various plant species and environments underscores their role as a sustainable and cost-effective solution for enhancing soil fertility and plant nutrition [38].

### Use of Phosphate-Solubilizing Fungi (PSFs)

Fungi are an essential component of soil microbial communities due to their high metabolic versatility and ability to access nutrients through the degradation of organic matter. In the context of phosphorus dynamics, PSFs significantly contribute to nutrient cycling and soil fertility. These fungi release extracellular enzymes and organic acids that dissolve insoluble phosphate compounds, making them available for plant uptake [39].

There are several fungal genera capable of solubilizing phosphorus that is unavailable in the soil and making it available to plants. Among the most efficient and commonly used genera are *Aspergillus*, *Penicillium*, *Trichoderma*, *Acremonium*, *Hymenella*, and *Neosartorya,* including in the commercial sphere [40].

Current knowledge regarding the diversity of arbuscular mycorrhizal fungi (AMF) is often focused on temperate ecosystems. Recent landscape-scale studies using high-throughput sequencing in tropical forests have revealed high turnover rates of AMF and significant environmental filtering driven by soil properties [41]. Furthermore, the stability of these species-rich communities is sustained by complex mutualistic networks. The integration of rare species into existing mycorrhizal networks including arbuscular, orchid, and *Cavendishioid mycorrhizae*, creates a nested network structure that stabilizes the ecosystem and facilitates resource distribution among diverse plant hosts [42].

The *Penicillium brevicompactum* strain inoculated into coffee plants increased the soluble phosphorus content in the soil in coffee plantations [43]. The use of phosphate-solubilizing fungi has been shown to release oxalic acid, which solubilizes phosphate and allows the formation of insoluble minerals such as pyromorphite and oxalate in a study with lead, thereby reducing Pb toxicity [44]. Also, PSFs improved the quality of arsenic-contaminated soil by increasing nutrients, enzymatic activities, and reducing arsenic uptake [45].

Several studies highlight the importance of phosphate-solubilizing fungi (PSF) at the soil–plant interface, demonstrating their essential role in agricultural sustainability. However, the high turnover of fungal taxa and their sensitivity to environmental filtration underscore that the loss of microbial diversity directly compromises soil structure and nutrient cycling [46]. These findings highlight that microbial interactions are not merely secondary processes, but rather essential mechanisms that regulate plant community structure and ecosystem variability [46]. This scenario reiterates the need for sustainable management practices aimed at preserving these vital symbionts, ensuring not only phosphorus availability but also the promotion of soil health and quality.

### Use of Phosphate-Solubilizing Actinobacteria and Cyanobacteria

Recent advances in agricultural biotechnology have highlighted the potential of endophytic microorganisms as biofertilizer to enhance plant growth while reducing dependence on agrochemicals. Among specialized groups of solubilizers, Actinobacteria and *Cyanobacteria* (PSAs) stand out due to their unique ability to colonize internal plant tissues without causing disease [46]. These endophytes actively participate in the phosphorus cycle by secreting organic acids and phosphatases, converting insoluble mineral phosphates into forms that are bioavailable to tropical host plants [46].

The conversion of primary forests into pastures in the Amazon profoundly alters the soil microbiota associated with the phosphorus (P) cycle [17]. Although deforestation increases the abundance of mineralizing groups, such as *Firmicutes* and *Cyanobacteria*, it reduces the functional diversity of the microbiome, however, the reestablishment of secondary forests demonstrates the ecosystem’s resilience, promoting the return of classes such as Proteobacteria (*Bradyrhizobiaceae* and *Beijerinckiaceae*) and the gradual restoration of solubilization genes [47]. This process allows microbial P-transformation functions to return to levels close to those of undisturbed forests, highlighting the importance of natural regeneration for the biome’s nutritional security.

A study using the cyanobacterium *Anabaena* sp. successfully observed an increase in phosphorus availability in the soil, showing promising potential for agricultural use and addressing future P shortages [48]. Microalgae and cyanobacteria have also been used to sequester and recycle phosphorus from effluents as slow-release biofertilizers, combining environmental remediation and nutrient recovery [49].

These bacteria can form symbiotic associations with plants and other microorganisms, and, because they are photosynthetic, they use carbon dioxide (CO₂) and water to produce monosaccharides and oxygen, which also benefits plants [50]. In agricultural environments, microalgae promote soil fertility and favor plant growth and protection, representing a promising alternative to dependence on chemical fertilizers and pesticides [51].

In the Amazonian context, the high diversity of these endophytes many of which remain unclassified represents a promising frontier for sustainable forestry. The ability of these microorganisms to establish stable endophytic populations inside tropical trees suggests that they can act as internal nutrient reservoirs, protecting the plant against phosphorus limitations typical of highly weathered acidic soils [17]. Furthermore, some species accumulate phosphorus in the form of polyphosphate, which favors their use as biofertilizers [25].

In short, the biotechnological potential of *Actinobacteria* and *Cyanobacteria* (PSAs) positions them as multifunctional agents in the sustainability of tropical ecosystems. The ability of these microorganisms to act as stable endophytes and internal nutrient reservoirs provides an important adaptive strategy for Amazonian trees in acidic and weathered soils.

## Dynamics of Plant-Microorganism Interactions and Growth Promotion

### Phosphate Solubilization and Mineralization Mechanisms

**Fig. 2:**
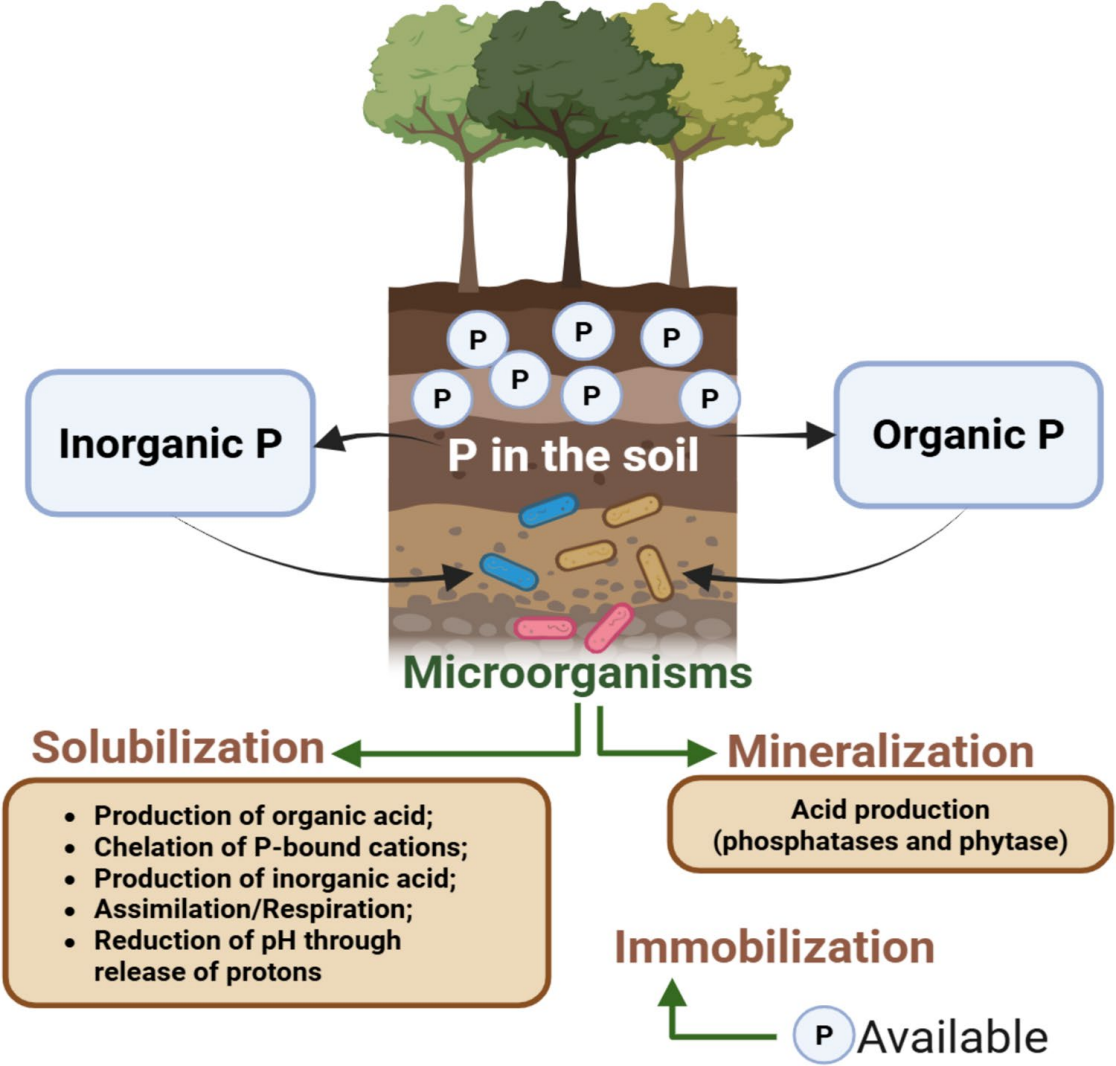
Schematic representation of the mechanism of solubilization/mineralization and immobilization of soil P by microorganisms. Created in https://BioRender.com

Phosphate-solubilizing microorganisms (PSMs) employ different mechanisms to make phosphorus (P) available, depending on the nature of the substrate [25]. The conversion of organic fractions occurs via mineralization, a fundamental step in the enzyme-mediated decomposition of organic matter. In contrast, the release of P from insoluble inorganic sources occurs through solubilization processes, generally driven by the secretion of organic acids [44]. Figure 2 illustrates a schematic representation of the phosphorus solubilization and mineralization processes mediated by soil microbiota.

**Fig. 3:**
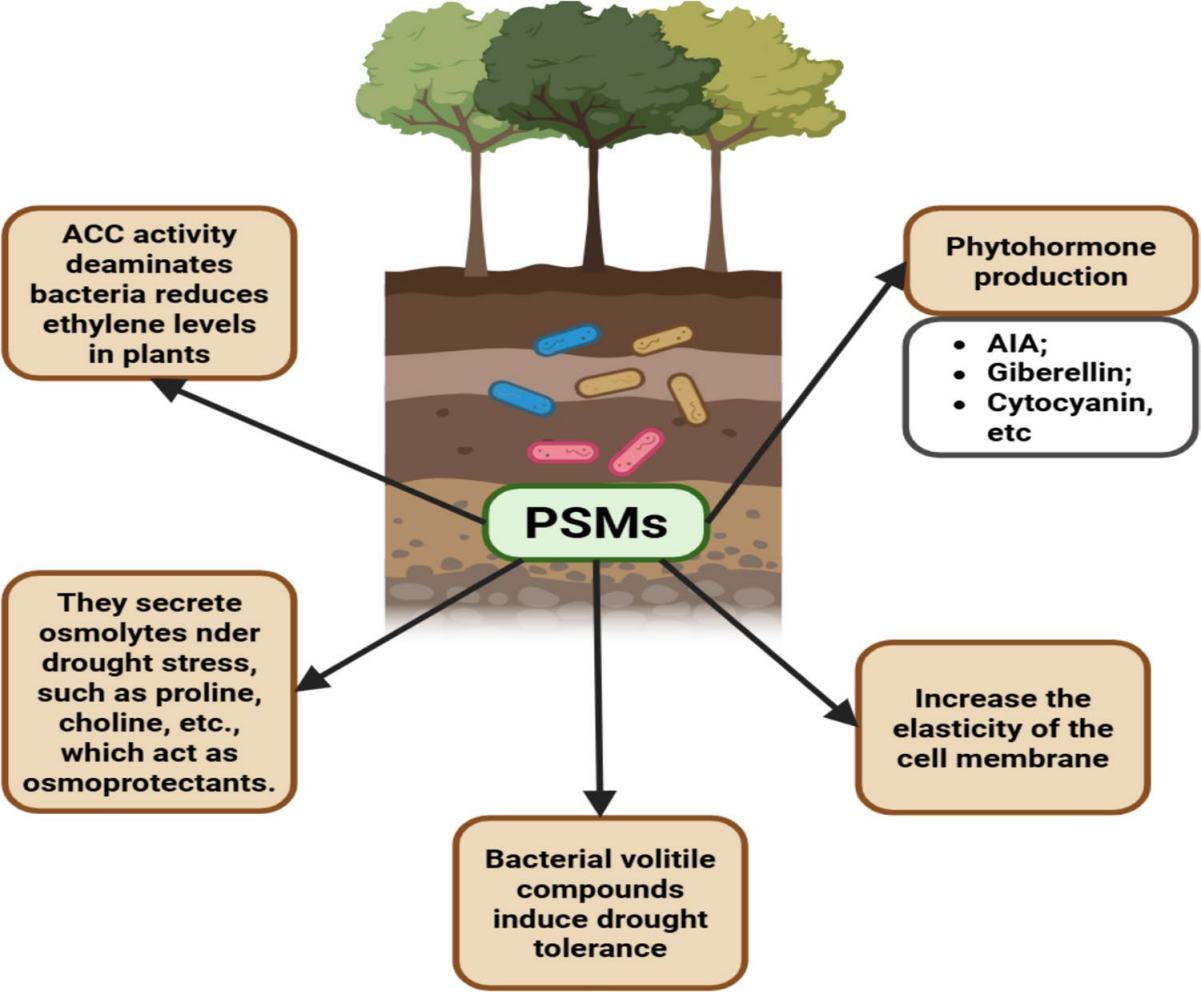
Synthesis of plant mechanisms of drought tolerance conferred by PSMs. (ACC: 1-aminocyclopropane-1-carboxylate). Created in https://BioRender.com

In summary (Fig. 3), these mechanisms occur through three pathways: chemical (acidification, chelation, proton exchange); biochemical/enzymatic (phosphatases, phytases); and biological (siderophores, AIA production (root expansion)).

#### Chemical Route

Acidification is the most common mechanism. Microorganisms (such as *Bacillus* and *Aspergillus*) secrete low-molecular-weight acids (e.g., gluconic, citric, oxalic, and succinic acids) and release protons [14]. These secreted protons lower the pH in the rhizosphere, promoting the dissolution of inorganic phosphate minerals, especially calcium phosphates [47]. Acidification is the exchange of protons (H^+^), during ammonium (NH_4_^+^) uptake, microorganisms release protons to maintain charge balance, which contributes to a drop in local pH (rhizosphere) and mineral solubilization [44].

The organic acids produced during solubilization lower the pH, increase the chelation of phosphorus-bound cations, and compete with phosphorus for adsorption sites in the soil, forming soluble complexes with metal ions (Ca, Al, Fe) and releasing phosphorus [52]. These organic acids are products of microbial metabolism, primarily through oxidative respiration or fermentation, when glucose is used as a carbon source [53].

Research in native forests and agroforestry systems [37] reveals high microbial diversity in *Bertholletia excelsa* roots, predominantly of the genus *Bacillus*. These isolates promote growth through nitrogen fixation (the *nifH* gene), indole compound synthesis, and aluminum phosphate solubilization key mechanisms for adaptation to acidic terra firme soils.

#### Biochemical Pathway

The mineralization of organic phosphorus is mediated by the action of phosphatases, enzymes that break ester bonds to release the free phosphate group [44]. The function of these enzymes depends on the optimal pH of the environment, classifying them as acid or alkaline phosphatases [54]. This enzymatic specialization allows the soil microbiota to adjust its metabolic strategy to optimize nutrient acquisition under varying physicochemical conditions [44].

Chronosequence studies in areas reforested with rubber trees (*Hevea brasiliensis*) in the Amazon demonstrate that as the plantation matures, the activity of acid and alkaline phosphatases recovers, reaching levels close to those of primary forests after 45 years [55]. In this scenario, phosphorus immobilized in microbial biomass establishes itself as the primary nutritional reserve for the crop, highlighting that the restoration of enzymatic functionality is critical for the sustainability of the phosphorus cycle [56].

Another important biochemical mechanism carried out by microorganisms is the production of phytase, enzymes specific for degrading phytate (phytic acid), which is the primary form of phosphorus storage in seeds and plant tissues that reach the soil [51].

#### Biological Pathway

As potent chelating agents, the siderophores produced by MSPs play a role in mobilizing inorganic iron [44, 57]. By competing for the metal cation that keeps phosphorus immobilized, these metabolites promote the chemical dissociation of phosphate minerals, this process not only meets the microbial cellular demand for iron but also makes soluble phosphorus in the rhizosphere available for plant uptake [44].

Studies have shown that PSMs secrete siderophores secondary metabolites with a high affinity for metal ions [58]. The chelation of these ions by siderophores leads to the formation of metal-siderophore complexes, which indirectly increases the availability of insoluble phosphate in the soil for plant uptake [25]. This mechanism represents an additional pathway through which PSMs improve phosphate nutrition in plants.

Root exudates from different plant species are released into the rhizosphere and consist of amino acids, sugars, flavonoids, and enzymes, providing vitamins, compounds, and hormones that sustain bacterial populations [59]. Auxin, ethylene, abscisic acid, cytokinins, and gibberellins are well-known phytohormones [4]. The presence of these phytohormones in root exudates influences cell proliferation, resulting in changes in the architecture of the root system through the formation of lateral roots and root hairs, ultimately improving the absorption of nutrients and water [60].

### Microbial Strategies for Promoting Plant Growth

Plant growth is coordinated by a complex exchange of molecular signals between the root and the aboveground parts, a process that is influenced by the soil microbiota [61]. This interaction establishes a signaling system in which plants provide exudates (sugars, organic acids, and vitamins) that serve as nutrients and signals for microorganisms [62]. In turn, free-living bacteria and fungi release classical phytohormones, such as auxins and cytokinins, as well as volatile compounds and molecules [61].

Most of the interaction between microorganisms and plants occurs in the rhizosphere microsystem. As the plant develops, the phenomenon of rhizodeposition occurs, which influences the attraction of beneficial microorganisms and the exclusion of harmful ones [63]. For this beneficial interaction to occur, microorganisms must be able to utilize root exudates for colonization, which allows them to proliferate rapidly [64]. They must also compete with the native microbiota.

Microorganisms can benefit plants in various ways, such as promoting growth through the mechanisms mentioned above and by increasing tolerance to abiotic stress [65]. The application of PSMs in agroforestry systems has emerged as an innovative and sustainable strategy, offering a promising alternative for optimizing plant productivity in resource-limited contexts and vulnerable environments [66].

In low-intensity systems, such as agroforests, the microbiota maintains a high abundance of genes involved in the active uptake of phosphorus (P), functioning similarly to native forests [67]. In contrast, in intensive monocultures, the application of mineral fertilizers shifts microbial metabolism toward biosynthetic genes, reducing dependence on organic mineralization [68, 69]. This genomic plasticity demonstrates that maintaining plant diversity in agroforestry systems is essential for preserving natural solubilization strategies and the resilience of the P cycle in the biome [69].

The Amazon rainforest’s response to increased CO_2_ depends on the plasticity of plants in acquiring phosphorus (P) [67]. In areas with greater P availability, the strategy focuses on physical acquisition (root expansion and mycorrhizae), while in poor soils (predominant in the region), the priority shifts to biochemical mining, based on the exudation of organic acids, acid phosphatase activity, and foliar reabsorption [62, 67]. This strategic alternation is what sustains primary productivity and ecosystem resilience under conditions of nutrient scarcity.

Beyond nutrient availability, inoculation has proven effective in consortia of native Amazonian microorganisms, specifically strains of *Trichoderma asperellum* and *Bacillus amyloliquefaciens*, where it has demonstrated to be an active strategy to accelerate this process. Inoculation with these strains not only increased total biomass but also optimized the physiological performance of seedlings, with significant increases in net CO_2_ assimilation, stomatal conductance, and transpiration [70].

### Determinant Factors in Plant-Microorganism Interactions

As demonstrated, microorganisms directly benefit their hosts by producing a variety of compounds that contribute to plant nutrition, and indirectly by protecting them against biotic factors (pests and pathogens) and abiotic factors (such as water deficit, salinity, and ionic stress) [71]. They associate with host plants by colonizing the rhizosphere and internal plant tissues. This colonization is driven by chemotaxis towards root exudates, which exhibit a certain specificity with receptors on the bacterial cell membrane [72].

In addition to nutrition, soil microbiota acts as a regulator of tropical biodiversity through density-dependent mortality mechanisms. Dense seedling germination alters the composition of Operational Taxonomic Units (OTUs) in the soil, creating microbial feedback loops that prevent the dominance of a single species [73]. In this context of biotic interaction, this microbial regulation also manifests itself symbiotically in crops of great socioeconomic relevance, such as guarana (*Paullinia cupana*). Inoculation with isolates of *Colletotrichum siamense* (infected by mycovirus) in guarana seedlings has been shown to severely reduce lesions caused by anthracnose. This protective effect is mediated by the activation of pathogenesis-related proteins (PRPs), with increases in the activity of enzymes such as peroxidase (POX) and phenylalanine ammonia-lyase (PAL) [74].

Soil is a heterogeneous environment that harbors a diversity of microorganisms in constant interaction, manifesting relationships of symbiosis, antagonism, mutualism, parasitism and saprophytism [75]. Among the various factors that can affect phosphate solubilization by bacteria are: competition with other soil microorganisms, the plant growth stage and the physicochemical properties of the soil, including organic matter content and pH [76].

PSMs from soils subjected to extreme environmental conditions, such as saline and alkaline, nutrient-poor soils or high-temperature regions, tend to solubilize more phosphate than those from temperate soils [4]. Other conditions that can influence the solubilization capacity or diversity of soil microorganisms include heavy metals, drought, flooding and light intensity, factors that alter the rhizosphere and affect microbial function, consequently influencing plant growth [77].

The presence of microorganisms becomes essential, as they perform osmoprotective and biological control functions in the rhizosphere environment [78]. They have also demonstrated the ability to tolerate and contribute to phytoremediation in soils where heavy metals have accumulated. These metals are harmful to plants, causing adverse effects on plant metabolism and contaminating the soil, which can make it unsuitable for cultivation [79].

Under conditions of high salinity, the root environment becomes desiccated, similar to what happens in situations of water scarcity. Changes in nutrient availability under these conditions impair photosynthetic activity and lead to an increase in ion absorption, thus promoting toxicity [80]. Under water deficit conditions, interactions between plants and microbes involve several mechanisms, including the production of phytohormones (abscisic acid, gibberellins, cytokinins and auxins), the synthesis of 1-aminocyclopropane-1-carboxylate (ACC) deaminase, which reduces ethylene levels, the production of antioxidants, the accumulation of compatible osmolytes, the production of exopolysaccharides and biofilms, the immobilization and solubilization of nutrients; and the production of siderophores (Fig. 3) [81].

The simultaneous occurrence of multiple stress factors affects the primary metabolism of plants, manifesting itself in disturbances in photosynthesis, compromising nutrient absorption, accelerating leaf senescence, cellular disorganization and reduced growth [66]. In this context, the adoption of techniques such as biofertilization emerges as a sustainable approach to optimize nutrient acquisition and, through its various mechanisms of action in the soil, mitigate the harmful effects of stress factors on plants [78].

A study on the combination of *Pseudomonas putida* and salicylic acid can mitigate water stress and improve plant growth [82]. *Bacillus subtilis* strains can confer tolerance to water stress in wheat and increase photosynthetic efficiency [83]. The combination of *Bacillus* sp. and the arbuscular mycorrhizal fungus *Rhizophagus clarus* promoted phosphorus uptake in plants under severe drought conditions [45].

Pioneer tree species, such as those of the genus *Handroanthus*, act as nuclei of biological organization by establishing highly complex rhizosphere bacteriomes distinct from the adjacent soil [84, 85]. Interactome modeling reveals that these pioneers support regulatory networks with greater molecular connectivity, where specific phylotypes coordinate the structure of the microbial community [85]. This functional specificity of the microbiome is crucial for the progression of the chronosequence, providing the necessary biological basis for ecological succession and the effective restoration of tropical forest cover.

Taking this into account, we highlight the main contributions of microorganisms that exhibit varied adaptability to abiotic soil and environmental conditions, such as salinity, drought, heavy metals and pH variations, preserving their metabolic activity and contributing to a sustainable environment [86].

## Use of PSMs

The use of inoculants is not new. In Brazil, research on biological nitrogen fixation (BNF) began in the 1950s with the pioneering work of agronomists Johanna Döbereiner (1924–2000) and Ruy Jardim Freire (1923–2015), which served as the basis for biotechnologies that currently use microorganisms [87]. This legacy consolidated the scientific basis for biotechnology to expand its focus to other limiting nutrients in tropical soils, such as phosphorus, through the selection of phosphate-solubilizing microorganisms (PSM).

Bioinoculants are living organisms that assist in the availability and absorption of nutrients by plant roots in the rhizosphere, especially by converting insoluble forms of organic and inorganic phosphorus into fractions assimilable by plants in the rhizosphere. They can also be defined as microbial inoculants, which are promising technologies that reduce the use of conventional inorganic fertilizers [88]. They are environmentally friendly organic products that contain specific microorganisms in concentrated forms, including fungi and bacteria derived from the rhizosphere soil, and can be effective and economically viable alternatives [89].

Recent trends towards ecologically and economically viable biotechnological efforts have driven the search for a new movement towards sustainable agriculture (Table 2). Sustainable agriculture is the most significant approach to counteract the decline in environmental quality, maintaining the extended ecological balance of environments [90].

**Table 2:** Representative phosphate-solubilizing microorganisms (PSMs), detailing their taxonomic group, species or isolate, ecological function, environment of occurrence, associated study species, mechanisms of action

Group	Species/isolate	Ecological function	Environment of occurrence	Study species	Mechanisms of action	References
Fungi	*Trichoderma harzianum FR-NST-009*	Phosphorus solubilizer	Soil	*Hevea brasiliensis*	Production of organic acids (mainly citric acid)	[95]
Fungi	*Trichoderma harzianum*	Assists in the initial growth of seedlings	Growing in pots	*Leucaena leucocephala (Lam) de Wit; Cedrela odorata L.; Albizia saman (Jacq.) Merr*	Production of plant hormones	[96]
Bacterias	*Azospirillum brasilense; Bacillus* sp.; *Azomonas* sp. *Azorhizophillus* sp.	Mitigation of abiotic stresses and promotion of growth	Growing in pots	*Tabebuia micrantha; Cabralea estrellensis*	Stimulates plant growth	[97]
Bacteria and fungi	*Pseudomonas orientalis; Chaetomium cupreum*	Act as biostimulants and bioremediators	Experimental field	*Eucalyptus globulus*	Production of plant hormones (AIA)	[61]
Bacterias	*Azospirillum brasilense;* *Bacillus* sp.	Rhizosphere biostimulant	Soil	*Cecropia pachystachya;Cariniana estrellensis*	Antioxidant enzymes; non-enzymatic antioxidant compounds and morphological parameters	[98]
Bacteria	*Enterobacter hormaechei* subsp. *xiangfangensis*	Rhizosphere biostimulant	Soil	*Pinus oocarpa*	Phosphorus solubilization	[99]
Bacteria	*Trichoderma* spp.	Rhizosphere biostimulant and biocontrol agent	Soil	*Theobroma cacao*	Promoting growth	[100]
Bacteria and fungi	*Peronospora viciae; Penicillium oxalicum; Paraburkholderia* sp.; *Burkholderia* sp.	Rhizosphere biostimulant	Soil	*Pinus massoniana*	Promoting seedling growth	[101]
Bacteria	*Bacillus wiedmannii; acillus paramycoides; Lysinibacillus fusiformis*	Rhizosphere biostimulant	Growing in pots	*Bertholletia excelsa*	Root biostimulation, increased survival, and promotion of initial growth	[102]
Bacteria	*Serratia* spp.; *Buttiauxella* spp.; *Bacillus* spp.	Rhizosphere and endophytic biostimulants	Soil	*Pinus montezumae; Pinus patula*	Production of indole-3-acetic acid (IAA), phosphorus solubilization	[103]
Bacteria	*Rhizobacteria*	Plant growth promotion and nutrient uptake enhancement	Nursery production / Amazonian nurseries	*Euterpe oleracea Mart*	Enhancement of photosynthetic efficiency (gas exchange and chlorophyll-*a* fluorescence), biomass accumulation, and leaf nutrient induction	[104]
Bacteria and Fungi	*phoD and phoX genes*	Organic phosphorus mineralization and mycorrhizal niche transition	Pasture-to-forest reforestation (19-year chronosequence)	*Pinus radiata, Eucalyptus nitens*	Replacement of arbuscular mycorrhizae by ectomycorrhizae, increasing net P mineralization. The abundance of phoD and phoX regulates alkaline phosphatase (ALP) secretion	[105]

### Integration of PSMs in Agroforestry Systems (SAFs)

The transitions from monoculture to Agroforestry Systems (AFS) significantly alters the structure of the microbial community and the functional diversity of Plant Growth Promoting Bacteria (PGPB) in the Amazon. Recent studies comparing cocoa (*Theobroma cacao*) monocultures with cocoa-açaí (*Euterpe oleracea*) AFS demonstrate that shaded and multispecies systems support distinct microbial profiles, highly sensitive to seasonal variations (dry season versus rainy season) [91].

The cultivation of açaí (*Euterpe oleracea*) in agroforestry systems favors the recruitment of rhizobacteria of the genus *Bacillus* (e.g., strains AP4-03, AP1-33, and AP2-36) with high biocontrol capacity. These isolates inhibit the growth of the phytopathogenic fungus Pestalotiopsis by up to 71% and produce indole-3-acetic acid (IAA) [92]. The presence of mechanisms such as solubilization of inorganic phosphates (15%) and organic mineralization (9%) in these agroforestry systems reinforces the potential of using microbial consortia to optimize plant health and vegetative vigor in the productive restoration of the Amazon.

Agroforestry systems (AFS) optimize ecological interactions and microbial biodiversity, functioning as refuges for soil conservation [93]. The tree complexity in these systems favors the formation of functional consortia (N_2_ fixers, P mobilizers, and biocontrol endophytes), reducing dependence on chemical inputs [93, 94]. Thus, managing plant diversity in AFS allows for the management of microbiota to provide ecosystem services and the sustainable restoration of degraded areas.

## PSMs as Allies in the Forest Restoration of Degraded Areas

MSVs are emerging biotechnological tools in agriculture and environmental engineering; however, significant gaps still exist in their use in forestry and the restoration of degraded ecosystems. In this context, changes in land use, such as the conversion of forest to pasture in the Amazon, alter the chemical properties of the soil, particularly pH, Al^3+^, total P, and labile P levels, and negatively impact the biodiversity of bacterial groups involved in P transformation processes and their potential functions, while the restoration of secondary forests can stimulate resilience [47, 106].

Given that ecological restoration significantly alters microbial functionality related to the phosphorus cycle, metagenomic studies in restoration chronosequences demonstrate a transition in P acquisition strategies: while the relative abundance of genes for organic P mineralization increases from 45.78% to 48.38% in advanced stages of succession, genes focused on inorganic P solubilization show a decline (from 27.19% to 25.03%) [107]. These data indicate that, as the ecosystem recovers, the microbial community led by *Proteobacteria* (up to 52.72%) and *Actinobacteria* prioritizes the cycling of accumulated organic matter, a fundamental mechanism for the resilience of tropical and subtropical forests in degraded soils.

The initial establishment of forest species in degraded areas represents one of the most critical stages in the ecosystem restoration process [108]. The germination and emergence phase of seedlings often faces severe edaphic constraints, such as low phosphorus availability, high acidity, organic matter deficiency, and the presence of toxic aluminum, which are common in highly weathered soils [78, 109]. This combination of factors requires integrated strategies ranging from the production of seedlings with high physiological vigor to management practices that favor the improvement of soil conditions in the first years [109].

It is important to note that the first ten years after planting are crucial for the success of forest restoration, as this is the period in which species are most vulnerable [110]. Interventions such as the use of strategic fertilization, management practices and growth-promoting microorganisms can accelerate initial growth [111]. Experimental evidence in Ultisols demonstrates that the addition of organic matter can increase P uptake by plants between 66.2% and 164%, an effect driven by the proliferation of phosphatase-producing copiotrophic bacteria, such as the genus *Klebsiella*, regulated by the input of labile carbon into the soil [54].

Soil biogeochemical processes can be restored more rapidly with the inoculation of specific groups of microorganisms [112]. Reinforcing this premise, metagenomic findings in reforestation chronosequences indicate that ‘functional genes of the phosphorus cycle, particularly pqqC and spoT, showed the greatest centrality in the network, indicating their dominant role in regulating nutrient dynamics’ by interconnecting the carbon (C), nitrogen (N), and phosphorus (P) cycles to increase soil fertility and microbial biomass (p < 0.01) [113]. Therefore, microbial biotechnology ceases to be merely a plant growth alternative and becomes the key component in the functional coordination of nutrient cycling in weathered soils.

Vivian et al. [113], conducted a review of advances in monitoring forest restoration, focusing on the role of soil microorganisms in forest development. The articles analyzed suggest a fluctuation in bacterial taxonomic diversity, with bacterial taxa adapted to adverse conditions being replaced by taxa capable of better exploiting the eutrophic conditions of the final stages of forest restoration. The mineralization of residual organic P is accelerated by the presence of labile carbon fractions (C-O-alkyl), which stimulate the activities of acid and alkaline phosphatases, regardless of the P content introduced by organic matter, consolidating microbial biotechnology as a key element in the recovery of fertility in highly weathered soils [109].

In this context, microorganisms with the potential to act in the phosphorus cycle should be more widely investigated, since most studies on the recovery of degraded areas have focused predominantly on nitrogen dynamics. Therefore, broadening the focus on the microbial phosphorus cycle in degraded areas is essential to promote more effective and sustainable restoration strategies, contributing to the restructuring of soil fertility and the establishment of more resilient forest communities.

## Challenges of Use of PSMs in Amazonian Forest Species

The Brazilian Amazon offers great potential for the isolation of plant growth-promoting microorganisms. In fact, several species of bacteria and fungi native to the Amazon have demonstrated potential for application in the cultivation of plants of agricultural importance [114]. Despite all the reported benefits of microorganisms in agricultural species, little is known about their effects on forest species.

In agroforestry systems in the Amazon, where soluble fertilizers are expensive, have high transportation costs and low purchasing power for producers, PSMs can be an economical alternative [93]. Unlike annual plants, trees need decades to grow and depend heavily on access to and recycling of nutrients and water from the soil [115]. Significantly, the diversity of planted trees can also improve the functional properties of the soil microbiota [116].

The lack of knowledge about the ecophysiological performance of native species growing in altered environments has become a limiting factor in recent years. Most of the consolidated data in the literature refers to the use of microorganisms in crops or in exotic species used in reforestation programs, with a scarcity of information applied directly to native forest species of the Amazon (Table 2). Although some progress has been made, the forest production system in the region still has potential for improvement through the application of plant growth-promoting bacteria [117]. [118], analyzed the plant growth-promoting potential of six selected bacterial strains. They found that the bacterium *Caballeronia sordidicola* LS-S2r could potentially be used as a comprehensive biofertilizer for boreal forest trees in highly disturbed and nutrient-poor soil conditions.

In Amazonian soils, especially degraded ones, the use of phosphate-solubilizing microorganisms can represent an even greater challenge. The composition of phosphate soils in the Amazon is complex and influenced by several factors, including phosphorus dynamics in the soil, reactions with other elements, the degree of weathering, and temporal variations [56, 119]. In addition, the intensity of land management modulates the biological response, since "microorganisms adapt by altering their strategies: prioritizing mineralization and solubilization or favoring biosynthesis, depending on the availability of P" [69]. In agroforestry systems, for example, the abundance of genera such as *Pseudomonas* and genes related to P acquisition suggests a maintenance of enzymatic functions close to those of the primary forest, contrasting with high-intensity monocultures [69].

Since the soil is typically acidic, Fe and Al oxides, as well as clay minerals, are primarily responsible for phosphorus adsorption. This limitation is critical, as direct evidence in primary forests indicates that approximately 60% of the Amazon basin has soils with such low phosphorus levels that net primary productivity is limited exclusively by this nutrient. Fertilization experiments demonstrate that the addition of P increases fine root productivity by up to 29%, suggesting that "phosphorus availability may restrict the responses of the Amazon rainforest to CO_2_ fertilization," with serious implications for the biome’s climate resilience [56].

Considering the available information on soil diversity in the Amazon, as well as information on the succession of microorganisms during reforestation, the use of PSMs based on individual strains can be challenging due to the enormous diversity of microorganisms associated with plants and soil and their intrinsic interactions [113], as well as the time elapsed between tree cultivation and maturity. A viable strategy to improve the availability of soluble phosphorus in the soil would be the use of synthetic microorganism communities (SynComs). This presents promising solutions to the problems faced by modern agriculture, resulting from climate change and soil degradation [120].

SynComs are one of the approaches used to assemble communities of microorganisms with reduced complexity to achieve a specific benefit. This strategy has been identified as an innovation in soil microbiology strategies for climate change mitigation and restoration of degraded areas [121]. The main advantage of using SynComs is that the construction of this community exploits a reductionist approach to take advantage of microbial benefits and understand multispecies interactions [101]. Synthetic communities (SynComs) involve the intentional co-cultivation of multiple microbial taxa under well-defined conditions that mimic natural microbiomes.

**Fig. 4:**
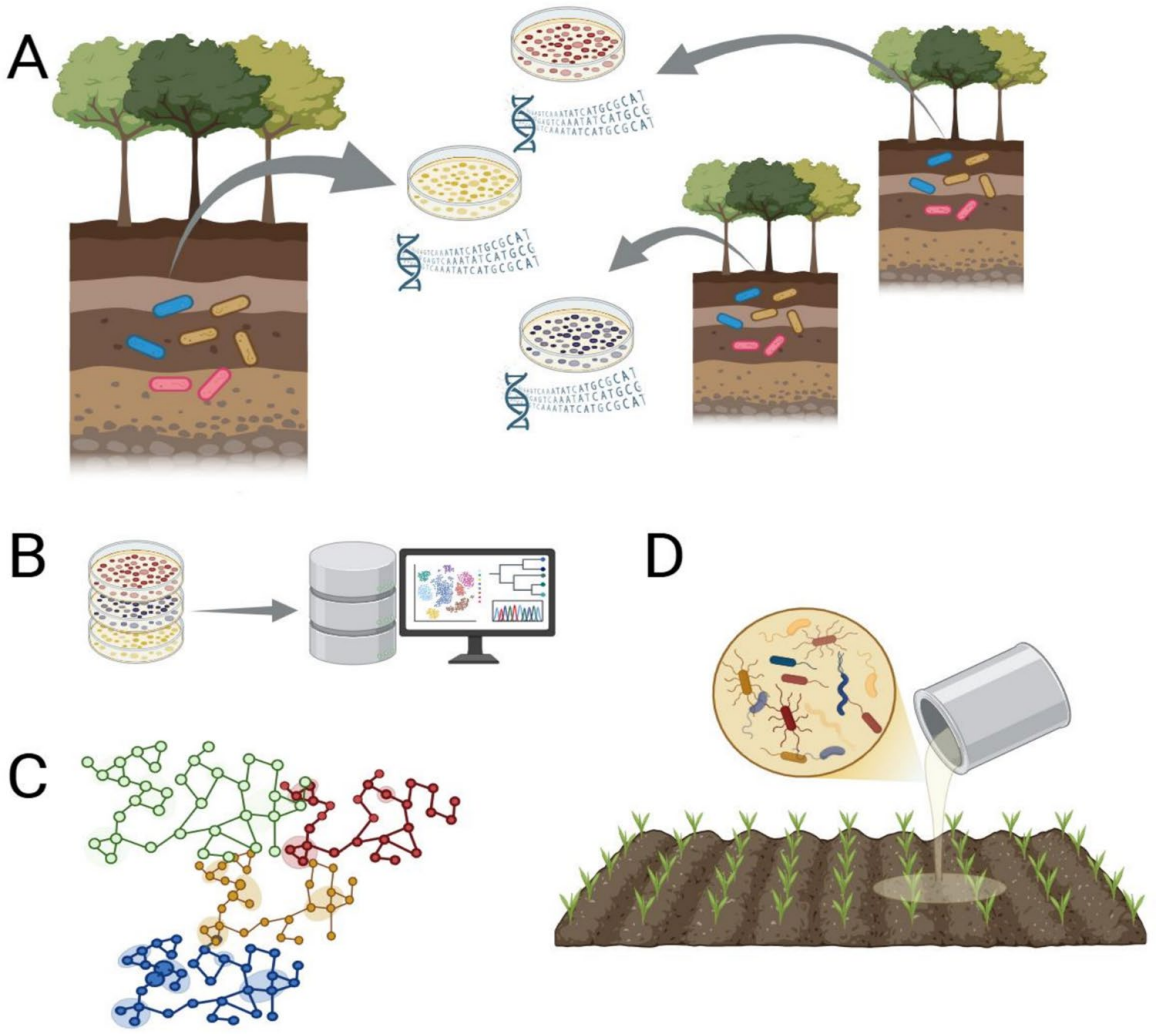
Workflow for the design of synthetic microbial communities (SynComs). (**A**) Isolation, sequencing, and preservation of microorganisms from soil. (**B**) Genomic characterization and functional profiling. (**C**) Network modeling of microbial interactions. (**D**) Assembly and application of SynComs for forest restoration. Created in https://BioRender.com

To design a synthetic community, community members are isolated from environmental samples, sequenced, and preserved in microorganism culture collections (Fig. 4A). After identifying the microorganisms and discovering their genomic potential, SynComs can be designed according to their taxonomic, functional, and ecological information [122] (Fig. 4B). In the case of phosphate-solubilizing microorganisms, a strategy for assembling consortia could be the use of systems biology tools to select non-competitive and cooperative microorganisms, which would allow the target microorganisms to be maintained in the soil for longer (Fig. 4C). Finally, SynComs can be assembled and applied in the reforestation of degraded areas (Fig. 4D).

Considering the possibility of using SynComs in reforestation areas, an urgent initiative, and the first step in the community project, is the isolation and sequencing of Amazonian microorganisms from different microbiomes. These isolates should be deposited in public banks and shared with researchers working in reforestation regions.

## Conclusions and Future Perspectives

The availability of phosphorus (P) in the soil remains the fundamental bottleneck for sustainable forest restoration in the Amazon. This review reaffirms that in highly weathered and nutrient-poor tropical soils, the use of Phosphate-Solubilizing Microorganisms (PSM) biofertilizers is not merely an alternative, but an essential ecological strategy. The integration of current findings demonstrates that transitioning from single-strain inoculants to Synthetic Communities (SynComs) enhances nutrient cycling by mimicking natural microbial interactions, thereby increasing the resilience of native species against nutritional stress.

The real-world impact of this approach extends beyond plant growth; it directly influences the carbon sequestration capacity of the Amazon biome. By "unlocking" legacy P fractions in the soil, these biotechnologies reduce reliance on high-cost, low-efficiency chemical fertilizers. This offers a scalable solution for recovering degraded areas and strengthening agroforestry systems, ensuring long-term productivity and the conservation of ecosystem services.

However, practical application in Amazonian forestry still faces limitations, such as the variability of microbial survival under field conditions and the scarcity of studies focused on native tree species. Future research must prioritize the isolation of region-specific microbiomes and the validation of innovative delivery technologies, such as carbon nanoparticles. It is imperative that future studies bridge the gap in understanding multi-species interactions in the soil to transform biotechnological potential into consolidated protocols for ecological restoration.

## Data Availability

The datasets generated during and/or analyzed during the current study are available from the corresponding author on reasonable request.
